# Incorrect Route for Injection: Inadvertent Tranexamic Acid Intrathecal Injection

**DOI:** 10.7759/cureus.13055

**Published:** 2021-02-01

**Authors:** Mustafa H AL-Taei, Mohammed AlAzzawi, Safa Albustani, Ghadier Alsaoudi, Eric Costanzo

**Affiliations:** 1 Internal Medicine, Jersey Shore University Medical Center, Neptune City, USA; 2 Pulmonary and Critical Care Medicine, Jersey Shore University Medical Center, Neptune City, USA

**Keywords:** tranexamic acid, spinal anasthesia, intrathecal complication, risk of seizure

## Abstract

Tranexamic acid has been increasingly used due to its safety and effectiveness. It has been associated with multiple reported cases of errors due to lack of attention, incorrect labeling of the syringes, or look-alike with other medications leading to the incorrect route of injection and the associated catastrophic sequela. Here we report a case of wrong route injection of tranexamic acid during spinal anesthesia, leading to myoclonic seizures and eventually intensive care unit admission of a patient undergoing orthopedic surgery. It is reported that higher doses of tranexamic acid would cause massive sympathetic discharge as evidenced by the initial hypertensive response reported in our case report and also in some repeated patient. Tranexamic acid induced seizures either from direct cerebral ischemia secondary to decreases in regional or global or from neuronal hyperexcitability by blockage of inhibitory cortical-gamma aminobutyric acid (GABA)-A receptors. Some evidence has been shown for dose-related neurotoxicity in the animal model, with greater severity and duration of seizure with increasing doses.

## Introduction

Tranexamic acid (TXA) is an antifibrinolytic; it is a lysine analogue, which exerts its effects through its interaction with plasmin and plasminogen in a dose-dependent manner. While at lower doses it is a competitive inhibitor of plasminogen into plasmin, at higher doses it is a non-competitive inhibitor of plasmin [1-2]. TXA, aprotinin, and aminocaproic acid have been used in multiple clinical procedures, including cardiovascular, obstetric, and orthopedic surgeries due to their effects in reducing blood losses [3]. TXA has been increasingly used due to its safety and effectiveness [3]. It has been associated with multiple reported cases of errors, due to lack of attention, incorrect labeling of the syringes, or look-alike with other medications leading to the wrong route of injection and the associated catastrophic sequela [3-4]. Here we report a case of wrong route injection of TXA during spinal anesthesia, leading to myoclonic seizures and eventually intensive care unit admission of a patient undergoing orthopedic surgery.

## Case presentation

A 76-year-old male presented with a past medical history of atrial fibrillation, who was admitted to the hospital for an elective replacement of his knee after a diagnosis of end-stage osteoarthritis was made. On presentation, the patient was alert, awake, oriented, and gave informed consent to proceed with the elective procedure. The patient’s vital signs were as follows: temperature, 98.9°F; heart rate, 84 beats per minute; blood pressure, 122/71 mmHg; and SpO2 98% on room air. The patient was taken to the operating room, and the procedure was aborted due to inadvertent injection of TXA intrathecally while administering spinal anesthesia due to the similarity of the two ampoules and lack of warning sign or coloring. The patient was having myoclonic seizures, vital signs as follows: blood pressure, 216/113; pulse rate, 89; respiratory, 36; oxygen saturation, 99%; he was treated with levetiracetam intravenous injection 1000 mg, midazolam intravenous infusion, and was intubated; blood pressure improved with sedation only; then the patient was admitted to the neurological intensive care unit (NICU). Magnetic resonance imaging (MRI) showed no acute abnormalities. Electroencephalogram (EEG) study performed was limited due to the patient’s sedation. The patient ICU stay was complicated by aspiration pneumonia and then ventilator-dependent respiratory failure, with worsening oxygenation, and he failed multiple proning attempts. The patient eventually passed away due to respiratory failure. A morbidity and mortality conference was presented about this case. Some changes are being implemented to guarantee that this kind of preventable error does not occur in most settings where these two medications are used.

## Discussion

TXA is an anti-fibrinolytic agent that forms a reversible complex that displaces plasminogen from fibrin resulting in inhibition of fibrinolysis; it also inhibits the proteolytic activity of plasmin. The majority of side effects are minimal and include mainly gastrointestinal upset [5], but neurotoxicity including seizures have been reported in post-marketing and in animal studies [6]. However, there is no information on using TXA intrathecally or reports of use to treat subarachnoid or intracranial hemorrhage in humans [6]. Few cases of accidental intrathecal injection have been reported regarding a wrong route injection of TXA. In a case reported by Wong et al., 1988, an 18-year-old patient undergoing appendectomy was injected with TXA intrathecally. The patient developed persistent sensory block of both lower extremities in addition to fever, myoclonus, urinary incontinence, and clonic convulsions, which then progressed to a generalized seizure that showed a good response to intravenous diazepam; the patient eventually fully recovered without residual neurological deficits [7].

In another case, an accidental intrathecal injection of TXA (75 mg) in a 30-year-old man was reported. The patient experienced a seizure, myoclonus, back pain, and hypertension that responded well to both clonazepam and phenobarbital. The patient got intubated due to refractory seizures and developed ventricular tachycardia, but eventually, symptoms resolved by day 4, and the patient was extubated [6]. A different case reported inadvertent intrathecal injection but a higher dose of TXA (500 mg) where the patient developed generalized convulsions and hypertension followed by refractory ventricular fibrillation and eventually cardiac arrest [8].

In our reported case, the patient presented with neurotoxicity due to the inadvertent intrathecal injection of TXA, which lead to myoclonus, seizures, and hypertension. The patient needed intubation and sedation to control the seizures; due to aspiration pneumonia, the patient remained ventilator-dependent and eventually passed away due to respiratory failure.

In conclusion, the exact mechanism by which TXA induces seizures is not fully understood. It is reported that higher doses of TXA would cause massive sympathetic discharge, as evidenced by the initial hypertensive response reported in our case report and also in some repeated patient [8]. TXA induced seizures either from direct cerebral ischemia secondary to decreases in regional or global [9] or from neuronal hyperexcitability by blockage of inhibitory cortical-gamma aminobutyric acid (GABA)-A receptors [10]. Some evidence has been shown for dose-related neurotoxicity in the animal model with greater severity and duration of seizure with increasing doses [11].

## Conclusions

In conclusion, the exact mechanism by which TXA induces seizures is not fully understood. It is reported that higher doses of TXA would cause massive sympathetic discharge as evidenced by the initial hypertensive response reported in our case report and also in some repeated patient. TXA induced seizures either from direct cerebral ischemia secondary to decreases in regional or global or from neuronal hyperexcitability by blockage of inhibitory cortical-GABA-A receptors. Some evidence has been shown for dose-related neurotoxicity in the animal model with greater severity and duration of seizure with increasing doses. An action is needed to make changes to the way in which ampoules are packaged to help reduce this major adverse event.
